# Cardioprotective Peptides from Dry-Cured Ham in Primary Endothelial Cells and Human Plasma: An Omics Approach

**DOI:** 10.3390/antiox14070772

**Published:** 2025-06-24

**Authors:** Clara Noguera-Navarro, Javier Stelling, Esteban Orenes-Piñero, Caterina Pipino, Francisco José Nicolás, Silvia Montoro-García

**Affiliations:** 1 Preclinical Research of Bioactive Compounds and Drugs (PREBIOF), Izpisúa Lab HiTech, Faculty of Health Sciences, Universidad Católica de Murcia (UCAM), Campus los Jerónimos, 30107 Murcia, Spain; cnoguera2@ucam.edu; 2 Regeneration, Molecular Oncology and TGF-ß, Instituto Murciano de Investigaciones Biosanitarias-Pascual Parrilla (IMIB-Pascual Parrilla), Hospital Clínico Universitario Virgen de La Arrixaca (HCUVA), 30120 Murcia, Spain; javier.stelling.ferez@gmail.com (J.S.); franciscoj.nicolas2@carm.es (F.J.N.); 3 Proteomic Unit, IMIB-Pascual Parrilla, Hospital Clínico Universitario Virgen de la Arrixaca, 30120 Murcia, Spain; eorenes@um.es; 4 Department of Biochemistry and Molecular Biology-A, University of Murcia, 30100 Murcia, Spain; 5 StemTeCh Group, Center for Advanced Studies and Technology-CAST (ex CeSI-MeT), Department of Medical, Oral and Biotechnological Sciences, University G. D’Annunzio Chieti-Pescara, 66100 Chieti, Italy; caterina.pipino@unich.it

**Keywords:** bioactive peptides, hydroxytyrosol, oxidative stress, cardiovascular health, ROS, endothelial dysfunction, omics analysis

## Abstract

Cardiovascular diseases are a leading cause of mortality, driving the search for alternative preventive strategies. This study investigates the antioxidant effects, among others, of a mixture of four bioactive peptides (BPs) derived from dry-cured pork ham on endothelial cells from healthy (C-HUVECs) and gestational diabetes (GD-HUVECs) pregnancies, as well as human plasma, using an integrative omics approach. Human umbilical vein endothelial cells (HUVECs) were treated with 300 μM purified BP, followed by transcriptomic and proteomic analyses. The results revealed significant alterations in mitochondrial gene expression and downregulation of genes associated with inflammation and oxidative stress in healthy HUVECs. Furthermore, BP treatment modulated key signalling pathways, including Ras and MAPK, leading to changes in the phosphorylation of ERK, AKT, and NF-κB, suggesting potential cardioprotective effects. The effects of BP were compared to those of the antioxidant hydroxytyrosol, highlighting their relative efficacy in vascular protection. The proteomic analysis of human plasma demonstrated BP-induced modulation of lipid metabolism, inflammation, and oxidative stress with notable changes in proteins such as APOA1 and MMP-8. These natural compounds demonstrate significant preventive potential in vascular health, highlighting their promise as effective tools for reducing cardiovascular risk before the progression of the pathology. These findings emphasize the importance of integrative omics in understanding the mechanisms behind BP’s effects and suggest promising applications for nutraceuticals aimed at cardiovascular protection.

## 1. Introduction

Cardiovascular (CV) diseases represent a major cause of mortality and morbidity globally, necessitating preventive strategies, including dietary interventions that provide bioactive compounds with versatile targets throughout the body [1]. Endothelial dysfunction is closely related to a higher risk of atherosclerosis, hypertension, diabetes, and dyslipidaemia. Indeed, endothelial cells serve as a suitable proxy for CV health in evaluating possible non-pharmaceutical strategies for the modulation of oxidative stress and inflammation pathways [2,3]. Recently, human fetoplacental endothelial cells from normal and gestational diabetes mellitus (GD) women have been used as a preclinical model of dysfunction to evaluate the efficacy of novel compounds such as anise and laurel essential oils [4]. Research has been traditionally centred around vegetal bioactive compounds, such as hydroxytyrosol (HT) or other potent antioxidant polyphenolic compounds [5,6].

Animal-derived sources have also proven to be a valuable reservoir of bioactive compounds, including bioactive peptides (BPs). These short amino acid sequences (2–20 residues) are involved in critical functions such as vascular tone and antioxidative regulation [7,8,9,10]. Moreover, BPs have garnered attention as a promising nutraceutical with CV protective effects, owing to their structural versatility and wide-ranging biological activities [11], such as antithrombotic, hypocholesterolemic, hypoglycaemic, antioxidant and anti-inflammatory effects, as reported in several clinical studies [12]. Recent research further supports their capacity to modulate key pathways related to CV health [13,14,15].

While the scientific evidence of the angiotensin-converting enzyme (ACE) inhibitory activity of BP from dry-cured pork ham is abundantly clear [16,17,18], and may explain the clinical benefits observed with regular consumption of BP-enriched ham [19,20], their broader mechanisms of action are not fully understood [9,21]. Recent in vitro studies have highlighted that BP can reduce inflammation [22] and platelet activation [23], thus supporting the existing clinical findings. However, the precise molecular mechanisms of how these peptides influence vascular endothelial function are still lacking. Advancing this knowledge requires the application of innovative methodologies—such as omics and advanced in vitro models—that can overcome the gap between molecular mechanisms and observed clinical improvements, ultimately enabling more targeted and effective therapeutic strategies.

In this study, we (i) employ an omics approach to efficiently profile their potential on responder cells, with relevant clinical impact, (ii) compare their effectiveness to HT as a gold standard for benchmarking the activity of BP across different cell conditions, and (iii) provide a proteomic analysis in human plasma to validate the model’s relevance for extrapolating in vitro findings to potential in vivo applications.

## 2. Materials and Methods

### 2.1. Isolation and Culture of Endothelial Cells

Primary Human Umbilical Vein Endothelial Cells (HUVECs) were collected immediately after delivery. Umbilical cord veins of newborns delivered between the 36th and the 40th gestational week were randomly selected from mothers affected by gestational diabetes (GD, n = 3) and healthy Caucasian mothers (C, n = 3) (Laboratory of Molecular Oncology and TGF-β, Hospital Universitario Virgen de la Arrixaca, Murcia, Spain) [24,25]. The cells were used in vitro for all experiments between the 3rd and 5th passages.

Umbilical cord veins were cannulated and perfused with 1 mg/mL collagenase type IA at 37 °C, and primary HUVECs were collected in an endothelial growth medium (HUVEC medium) composed of DMEM/M199 (1:1) supplemented with 1% L-glutamine, 1% penicillin/streptomycin, and 20% fetal bovine serum (FBS). Then, the cell suspension was centrifuged at 1200 rpm for 10 min and the cell pellet was resuspended in a HUVEC medium and plated on 1.5% gelatin-coated tissue culture plates (Sigma-Aldrich, St. Louis, MO, USA) in a complete low-glucose (1 g/L) DMEM and M199 medium (ratio 1:1) supplemented with 20% FBS, 10 μg/mL heparin and 50 μg/mL endothelial cell growth factor, 1% penicillin/streptomycin, and 1% L-glutamine (standard medium) according to previous studies [2]. After a 24 h pre-incubation period in the standard medium, it was replaced by a medium with 10% FBS medium supplemented with 300 μM of purified BP (BP1: KPVAAP; BP2: KAAAATP; BP3: KPGRP; BP4: AAATP mixture) [22] or 100 µM HT. Hydroxytyrosol is used as a positive control (or reference compound) for assessing the relative effectiveness of BP.

After 24 h, cells were serum-starved (0.1% FBS) for 2 additional hours and later stimulated with 1 ng/mL Tumour Necrosis Factor-α (TNF-α) supplemented with 300 μM synthetic BP or 100 µM HT for 2, 6, and 24 additional hours, according to previous studies [22]. For each bioactive compound, the experiments were carried out on three different cellular strains of each phenotype (C-HUVECs and GD-HUVECs), each in technical triplicate.

### 2.2. In Vitro Studies and Bioactive Compound Treatments

The sequence, protein origin, and ACE inhibition activity of four peptides from dry-cured ham (BP1, BP2, BP3 and BP4) were characterized by our group in a previous study [22]. These peptides were synthesized chemically by GenScript Corporation (Piscataway, NJ, USA) at the highest purity certified using liquid chromatography/mass spectrometry (LC-MS) analysis. Hydroxytyrosol was purchased from Sigma-Aldrich.

### 2.3. Western Blot

Human cells were washed with cold PBS and lysed using RIPA buffer (Sigma-Aldrich, St. Louis, MO, USA) supplemented with phosphatase inhibitors (I and II) and protease inhibitors (all from Sigma-Aldrich, St. Louis, MO, USA). Protein concentration of the protein lysates was determined using the Bradford Protein Assay. Protein samples (30 µg) were loaded into a sodium dodecyl sulfate–polyacrylamide gel electrophoresis (SDS-PAGE) and then transferred to membranes. The membranes were blocked with 5% milk, followed by immunoblotting with anti-NF-κB (1:1000) primary antibody. In parallel, other membranes were blocked with 5% BSA, followed by immunoblotting with the following primary antibodies: rabbit anti-*p*-NF-κB (1:1000), *p*-ERK (1:2000), ERK (1:1000), *p*-AKT (1:2000), and AKT (1:500) overnight at 4 °C, followed by rabbit horseradish peroxidase-conjugated secondary antibody (1:1000) (Santa Cruz Biotechnology). The immune complexes were visualized using the ECL Plus detection reagent (Thermo Scientific, Rockford, IL, USA) in a ChemiDoc MP (BioRad, Hercules, CA, USA). β-actin was also immunoassayed by primary antibody mouse monoclonal-β-actin (1:4000) for 10 min and then fluorescent goat anti-mouse (1:2500). The resulting ratio density protein/β-actin was considered as an index of protein expression in arbitrary units. For each bioactive compound, the experiments were carried out on three different cellular strains of each phenotype (C-HUVECs and GD-HUVECs), each in technical triplicate.

### 2.4. RNA Isolation and Quantitative PCR

RNA extraction from HUVEC assays was extracted using the RNeasy-mini system (Qiagen, Venlo, The Netherlands). RNA concentration was measured using a NanoDrop (ND-1000; NanoDrop Technologies, Wilmington, DE, EE. UU), and 1 µg was retro-transcribed using iScript reagents (Bio-Rad, Hercules, CA, USA). The resulting cDNA was used for quantitative PCR (qPCR), implementing the SYBR premix Ex Taq kit (Takara Bio Europe/Clontech, Saint-Germain-en-Laye, France) according to the manufacturer’s instructions. Gene expression levels of vascular cell adhesion molecule-1 (*VCAM-1*), *TNF-α*, and Mitochondrially Encoded 12S RRNA (*MT-RNR1*) were normalized to the glyceraldehyde 3-phosphate dehydrogenase (*GAPDH*) content of each sample by applying the comparative target gene quantification cycle Cq method (2−∆∆Cq). The experiments were carried out on two different strains for C-HUVECs and two different strains for GD-HUVECs, each in technical triplicate.

### 2.5. RNA Sequencing and Analysis

RNA sequencing was performed by GenomeScan BV (Leiden, The Netherlands). All RNA samples were polyA-enriched and sequenced with a 150-bp paired-end read length with a sequencing depth of 20 million reads on a NovaSeq6000 platform (Illumina, San Diego, CA, USA, EE. UU). The quality and yield were measured with the Fragment Analyzer (Advanced Analytical Technologies, Heidelberg, Germany). Samples with RNA quality numbers ≥ 6 were selected for RNA library preparation in an ISO/IEC 17025-accredited protocol (TruSeq RNA library preparation kit v2, Illumina, San Diego, CA, USA). During library preparations, mRNA was enriched using oligo (dT) magnetic beads and subsequently fragmented. First-strand cDNA synthesis was performed using random primers, followed by adapter ligation and PCR amplification. Sequencing generated raw data underwent image analysis, base calling, and quality check using the Illumina data analysis pipeline Real-Time Analysis 2.4.11 and Bcl2fastq 2.17.

The RNAseq reads were aligned to the human genome reference assembly GRCh37.75. Post-alignment, the raw sequencing data were normalized using the fragments per kilobase of exon per million mapped fragments (FPKM) method to adjust for sequencing depth and gene length variability. Differential gene expression analysis was performed using the DESeq2 package (https://bioconductor.org/packages/release/bioc/html/DESeq2.html (accessed on 17 July 2023)). For differential gene expression (DGE) analysis, read counts were loaded into the DESeq2 version 1.14.1 within the R platform version 3.3.0. This analysis identified DEGs between predefined sample groups. The DEGs were then subjected to gene regulatory pathway enrichment analysis using three separate tools: Gene Ontology (GO) Enrichment Analysis, KEGG Pathway Analysis, and Reactome Pathway Analysis.

### 2.6. Human Plasma Samples

Human plasma samples were selected from a previous clinical study with participants at moderate CV risk [19]. Inclusion criteria of the patients were the following: aged 20–65 years; 130 ≤ systolic blood pressure ≤ 150 mmHg; diastolic blood pressure > 80 mmHg (home blood pressure, average of 3 readings taken after 5 min rest); body mass index (BMI): 20.0–35.0 kg/m^2^; basal cholesterol level > 200 mg/dL and/or basal glucose level > 100 mg/dL or HbA1c among 6.5–7.5. Informed consent was signed by all the participants [19]. Participants consumed 80 g of dry-cured pork ham enriched in BP daily for 28 days. The BP mixture was characterized in vitro and displayed angiotensin I-converting enzyme (ACE-I) and 3-hydroxy-3-methyl-glutaril-CoA reductase (HMG-CoAR) inhibition activity [19]. The BPs were characterized in vitro and displayed ACE-I and HMG-CoA reductase inhibition activity [10].

The study protocol was approved on 24 November 2017 by the UCAM Ethics Committee and Clinical Research Ethics Committee of the Servicio Murciano de Salud (Area 1, Región de Murcia, Spain), and was conducted under the Declaration of Helsinki. This trial was registered in November 2021 at ClinicalTrials.gov (Identifier CE111703).

### 2.7. Plasma Treatment and Protein Extraction

Blood extraction was developed at two different points: before and after dry-cured pork ham consumption (n = 30 patients). Citrated blood samples were immediately centrifuged at 2.500× *g* for 15 min. Then, the plasma was aliquoted into a 1.5 mL tube and frozen in a −80 °C freezer until needed.

Protein quantification from plasma samples was performed using the Pierce BCA protein Assay Kit (Thermo Scientific, Rockford, IL, USA). SDS-PAGE was performed at room temperature using 10% acrylamide resolving gel and 5% acrylamide stacking gel. Proteins within gel bands were first reduced and alkylated using dithiothreitol (DTT) and iodoacetamide, respectively, and then digested to peptides by trypsin proteomics grade (Sigma-Aldrich, St. Louis, MO, USA, EE.UU.).

### 2.8. Liquid Chromatography in Reverse Phase Coupled to Mass Spectrometry (LC-MS)

Tryptic peptides were analysed by liquid chromatography/mass spectrometry (LC-MS). The column, BioBasic-15, 5 µm particles, 300 Å pore size, 0.18 mm ID-30 mm L (Thermo, San Jose, CA, USA), was connected to a Surveyor MS Pump Plus (Thermo, San Jose, CA, USA). Mobile phase A was 0.1% formic acid in water, and B was 0.1% formic acid in methanol. The ion trap MS was operated in a data-dependent MS/MS mode where the five most abundant peptide molecular ions in every MS scan were sequentially selected for collision-induced dissociation with a normalized collision energy of 34%. Dynamic exclusion was applied to minimize the repeated selection of peptides previously selected for collision-induced dissociation.

### 2.9. Database Searching

All LC-MS samples were analysed using Sequest (Thermo Fisher Scientific, San Jose, CA, USA) version IseNode in Proteome Discoverer 2.3.0.523 and X! Tandem (version 2017.2.1.4). Sequest was set up to search uniport homo-sapiens filtered organism (163,787 entries) after digestion with the trypsin enzyme. X! Tandem was searched with a fragment ion mass tolerance of 1.00 Da and a parent ion tolerance of 1.00 Da. Sequest was searched with a fragment ion mass tolerance of 1.2 Da and a parent ion tolerance of 1.5 Da.

Scaffold (version Scaffold_4.10.0, Proteome Software Inc., Portland, OR, USA) was also used to validate LC-MS/MS-based peptide and protein identifications. Protein identifications were accepted if they could be established at greater than 99% probability and contained at least 1 identified peptide. Protein probabilities were assigned by the Protein Prophet algorithm. Proteins that contained similar peptides and could not be differentiated based on LC-MS analysis alone were grouped to satisfy the principles of parsimony. Proteins sharing significant peptide evidence were grouped into clusters.

### 2.10. Bioinformatics Analysis of Proteomics Data

The identified proteins were further analysed using several bioinformatics tools to elucidate their biological significance and involvement in metabolic and signalling pathways. GO Annotation: The identified proteins were annotated for their biological processes, molecular functions, and cellular components using GO analysis. This provided insights into the functional roles of the proteins and their involvement in various biological processes.

Pathway Analysis: Identified proteins were mapped to known metabolic and signalling pathways using the Kyoto Encyclopedia of Genes and Genomes (KEGG) and Reactome pathway databases. This mapping helped in understanding the pathways modulated by bioactive compounds consumption, particularly those related to lipid metabolism and inflammatory responses.

Protein–Protein Interaction Networks: Interaction networks were constructed using tools like STRING (https://string-db.org/, accessed on 4 September 2023) and Cytoscape (https://cytoscape.org/, accessed on 4 September 2023) to visualize the interactions between identified proteins. This helped in identifying key hub proteins and understanding the complex interplay between different proteins in the context of the observed biological effects.

Quantitative Analysis: Label-free quantification methods were employed to assess the differential expression levels of proteins between sample groups. This quantitative analysis provided insights into the proteins significantly upregulated or downregulated post-bioactive peptide consumption.

### 2.11. Enzyme-Linked Immunosorbent Assay (ELISA)

Detected proteins were measured in plasma samples using different ELISA kits following the manufacturer’s instructions at different dilutions: 100,000-fold for APOA2, 1,000,000-fold for APOA1, 20,000-fold for APOB, and 20-fold for MMP-8 (Thermo Fisher Scientific, Carlsbad, CA, USA).

### 2.12. Functional Enrichment Analysis

The STRING database was used to integrate all known and predicted associations between genes, including both physical interactions as well as functional associations [26]. Functional enrichment analysis for proteomics data was performed using FunRich (http://www.funrich.org/, accessed on 16 January 2024) [27]. Briefly, the quantitative proteomics list was imported into the FunRich tool and cellular components, biological processes, molecular functions, and enriched biological pathways were identified for these proteins.

### 2.13. Statistical Analysis

Statistical analysis was conducted using GraphPad Prism 10, SPSS analysis software program (v22, IBM, Armonk, NY, USA), and R 4.3.2. Normality of continuous variables was assessed with Shapiro–Wilk tests, and data are presented as mean ± SD. In in vitro experiments, gene expression (ΔΔCt) and densitometric Western blot values from C-HUVECs were compared to GD-HUVECs using two-tailed unpaired Student’s *t*-tests for normal distributions or Mann–Whitney U tests, as appropriate. Transcriptomic counts were processed in DESeq2, and proteomic label-free intensity values in Sequest and Scaffold. ELISA results from paired pre-/post-intervention plasma samples were analysed with Wilcoxon signed-rank tests. Transcriptomic counts were processed in DESeq2, and proteomic label-free intensity values in Sequest and Scaffold. Functional enrichment of significant proteins was explored using clusterProfiler (GO, KEGG, Reactome, Toronto, ON, Canada), and protein–protein interaction networks were examined in STRING v12.0 with a confidence score ≥ 0.70; network topological parameters were compared using permutation tests (n = 10,000). Exact *p*-values are provided in the text or figures, with values *p* < 0.05 considered statistically significant.

## 3. Results

### 3.1. Impact of Bioactive Peptides and Hydroxytyrosol on Inflammatory and Oxidative Pathways in Healthy and Diabetic HUVECs

**Figure 1:**
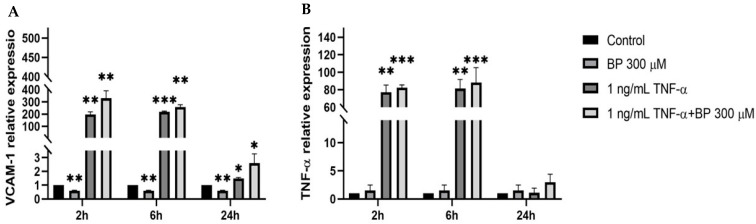
Expression of inflammatory markers measured by RT-qPCR after 2, 6, and 24 h of 300 µM BP, and/or 1 ng/mL TNF-α in C-HUVECs. (**A**) *VCAM-1* and (**B**) *TNF-α* levels. Fold induction for each gene was calculated versus the control at the corresponding time point. Results represent the average ± SD of three independent experiments. Statistical significance was determined via ANOVA followed by Tukey’s multiple comparison test. (*) Significantly different from control. *: *p*-value < 0.05; **: *p*-value < 0.01; ***: *p*-value < 0.005.

To investigate the effects of BP on inflammation and oxidative stress, we assessed the expression levels of key inflammatory markers such as *TNF-α* and *VCAM-1* in C-HUVECs. Various time intervals (specifically 2, 6, and 24 h) were analysed to identify the time window where the anti-inflammatory effects were most pronounced (Figure 1). In C-HUVECs, BP led to a moderate decrease in *VCAM-1* expression at 2, 6, and 24 h (Figure 1), whilst BP treatment was unable to reverse the TNF-α effect. After 24 h of TNF-α, the lower response could be indicative of either TNF-α-induced cytotoxicity or a desensitization/adaptive response to prolonged exposure It was observed that the antioxidant and anti-inflammatory response was most discernible at 6 h, which was further confirmed by incubating C-HUVECs with 100 µM HT (Supplementary Figure S1). As a positive control, a non-cytotoxic dose of HT was able to reverse the 6 h pro-inflammatory and oxidant effect of TNF-α.

**Figure 2:**
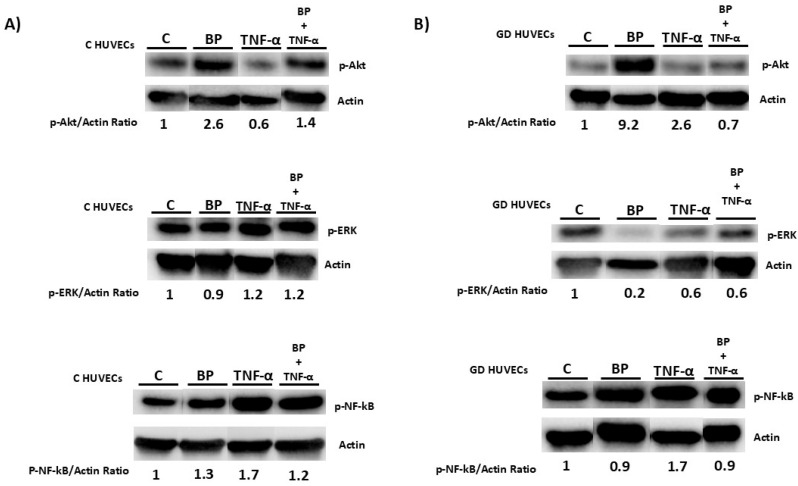
Protein expression levels of *p*-AKT, *p*-ERK, and *p*-NF-kB were analysed by Western blot in (**A**) C-HUVECs and (**B**) GD-HUVECs after 1 ng/mL TNF-α treatment for 6 h and/or 300 µM BP. The ratio of densitometries of *p*-AKT, *p*-ERK, and *p*-NF-kB to β-actin expresses the relative quantitative expression. The Western blot has been manually cut to show these specific bands.

Western blot analyses showed that there was a significant enhancement in the phosphorylation states of protein kinase B (*p*-AKT) following BP treatments in C-HUVECs and GD-HUVECs, as evidenced in Figure 2. Phosphorylation of Extracellular Signal-Regulated Kinase (*p*-ERK) was found to be decreased in BP-treated GD-HUVECs as well (Figure 2B), whilst the addition of TNF-α + BP did not recover the original status, as similarly observed using the qPCR method. On the contrary, *p*-NF-κB was barely reverted in C- and GD-HUVECs in TNF-α supplemented with BP (Figure 2). The total forms of NF-κB, ERK, and AKT did not exhibit significant changes, underscoring the specificity of the BP’s action on their phosphorylated counterparts.

Western blot analysis confirmed that HT treatment enhanced *p*-AKT (Supplementary Figure S2A) and *p*-ERK (Supplementary Figure S2B), which are associated with anti-inflammatory pathways. Notably, this anti-inflammatory effect was more pronounced in C-HUVECs than in GD-HUVECs, suggesting a reduced sensitivity of diabetic endothelial cells to HT’s protective effects, as well as it being found in the presence of BP. Overall, these results indicate that BP and HT show differential responsiveness, being lower in GD-HUVECs.

### 3.2. Differential Gene Expression Analysis Induced by BT and HT

A detailed differential gene expression (DGE) analysis to identify specific genes modulated by BP and HT in C-HUVECs and GD-HUVECs was performed. DGE analysis using DESeq2 identified a distinct expression profile after 6 h of incubation for each condition, revealing significant differences between the cellular responses to BP and HT, especially when comparing healthy to diabetic cells.

When C-HUVECs were exposed to BP, we identified 22 DGE that withstood the rigour of multiple testing corrections (a comprehensive list of all DGE can be found in Table 1). Among these, 17 genes exhibited reduced expression, whereas 5 showed increased expression levels following BP treatment compared to control conditions. The upregulated genes notably included those encoding mitochondrial ribosomal RNAs, *MT-RNR1* and *MT-RNR2*, as well as *MMP-1*. Conversely, genes such as *TGF-βI*, *PTX3*, *SULF1*, DSP, SELE, *ANXA8*, and *ILF44I* were among the downregulated entities (Table 1). On the other hand, HT treatment led to the upregulation of 14 genes with roles in oxidative stress response, mitochondrial function, and anti-inflammatory pathways. *MT-RNR1* and *MT-RNR2* displayed comparable regulation patterns in response to both bioactive compound treatments (Supplementary Table S1). Among these, eight genes exhibited increased expression, whereas six showed decreased expression levels following HT treatment compared to control conditions. Conversely, genes such as SOX18 and different histones such as *H1–3*, *H2BC18*, *H3C12*, and *H3C3* were downregulated (Supplementary Table S1). 

When comparing the gene expression profile of control C-HUVECs and GD-HUVECs, 36 DGE were significantly expressed. Genes of interest such as *FGF5*, *RPS4Y1*, *CCNA1*, *CCBE1*, and *PRRX1* were significantly increased in GD-HUVECs, whilst other genes such as *CLCE14*, *AIF1L*, *TBX1*, *AUTS2*, and *EBF3* were decreased.

Hence, GD-HUVEC cells exposed to BP resulted in the identification of two genes when compared to unstimulated conditions: upregulation and downregulation of *MT-RNR1* and *H3C10* histone, respectively. As *MT-RNR1* expression appeared in healthy and pathological cell lines, the increase in *MT-RNR1* expression in the presence of BP in C-HUVECs and GD-HUVECs was further confirmed by qPCR (*p* = 0.025 and *p* = 0.008, respectively). Accordingly, with a lower expression of genes, HT treatment resulted in a narrower transcriptional response in GD-HUVECs, with fewer upregulated genes (7 DGE), including *STC1*, *HMOX1*, *NR4A1*, and *SOCS1*, among others (Supplementary Table S1). 

In order to further explore the potential for improving a pathological condition, the impact of BP was assessed in the presence of TNF-α. Initially, it was found that TNF-α treatment alone induced alterations in 205 genes in C-HUVECs, including upregulation of *CX3CL1*, *VCAM-1*, *TRAF1*, *ANO9*, *TNFRSF9*, and *SELE*. These findings suggest that the control cells were responsive to treatments, making them suitable for further analysis. A comparison analysis in C-HUVECs between TNF-α-and TNF-α + BP demonstrated that the combined BP treatment failed to restore gene expression levels to those observed in the basal state in the control group. Conversely, in healthy cells, the comparison analysis between TNF-α, and TNF-α + HT revealed that the combined treatment significantly reduced the expression of inflammatory and oxidative genes, including *SELE*, *BIRC3*, *TRAF1*, *VCAM1*, *ICAM1*, *CXCL8*, *NR4A3*, and *NFKB2*, among others. These findings indicate that while BP treatment appears to be more effective in non-pathological conditions, it is unable to reverse an established oxidant and inflammatory state. In contrast, HT exhibits a greater potential as an antioxidant or anti-inflammatory compound due to its ability to attenuate the inflammatory state in C-HUVECs. These results suggest that BP remains important in prevention treatments rather than in pathological treatments.

TNF-α stimulation in GD-HUVECs resulted in the identification of 116 DEGs, including the upregulation of *BIRC3*, *TRAF1*, *TNFAIP3*, *VCAM1*, *CXCL8*, *RELB*, *CXCL2*, *CSF1*, *IL32*, *CXCL1*, *TNIP3*, and *NR4A3*, among others. Moreover, comparison between TNF-α treatment alone and TNF-α + BP did not produce any relevant results in GD-HUVECs (Supplementary Figure S3). Notably, when this pathological cell line was subjected to TNF-α alone with TNF-α + HT, only 21 DEGs were identified, including the upregulation of *HMOX1*, *STC1*, *EGR1*, and *DUSP5*, among others. Potential inflammatory and oxidant genes, such as VCAM-1 and IL6, were downregulated with TNF-α + HT treatment. However, neither treatment was able to revert the inflammatory state in GD-HUVECs to the baseline levels observed in the control condition. Thus, our GD-HUVECs serve as an in vitro endothelial model, confirming that bioactive compounds (BP and HT) are not entirely suitable for treating pathological inflammatory and oxidant states. Nonetheless, under healthy conditions, these compounds, particularly BP, can be employed as preventive tools.

### 3.3. Proteomic Analysis of Plasma Samples Following BP Consumption

Proteomic profiling using liquid chromatography/tandem mass spectrometry (LC-MS) identified several proteins associated with lipid metabolism and CV health that were modulated post-consumption of BP-enriched dry-cured ham.

The Sequest software (version IseNode in Proteome Discoverer 2.3.0.523) determines the qualitative presence of these peptides but cannot delineate the precise up- or downregulation of protein expression following consumption of dry-cured pork ham BP. However, the ScaffoldQ software (version Scaffold_4.10.0) provides a semi-quantitative assessment, revealing notable differential expressions in protein levels.

Among the 72 proteins identified (Table 2), Apolipoprotein A1 (APOA1) and Apolipoprotein A2 (APOA2) showed significant changes in expression, with APOA1 levels increased and APOA2 levels decreased following BP intake. These apolipoproteins play essential roles in lipid transport and cholesterol regulation, supporting the cardioprotective potential of BP. Additionally, Matrix Metalloproteinase-8 (MMP-8), associated with inflammatory responses, showed a reduction in expression, further aligning with BP’s potential role in moderating inflammation.

The in vivo proteomic results corroborate the transcriptomic findings in C-HUVECs, where lipid metabolism and inflammatory markers were modulated by BP, albeit with more pronounced effects in the clinical samples than in the GD-HUVECs. This alignment between in vitro and in vivo data underscores the translational potential of the cellular model for predicting real-world impacts of bioactive compounds on CV health.

Later, ELISA assay evaluations were conducted by diluting the plasma samples in various concentrations. The analysis revealed that the levels of apolipoproteins, specifically APOA1 and APOA2, did not show significant differences. Furthermore, APOB was also assessed, though it did not display notable changes, to ascertain the APOB/APOA1 ratio. Notably, the administration of BP was associated with a statistically significant decrease in the APOB/APOA1 ratio (*p* = 0.018), which contributes to a decrease in CV risk. Additionally, there was a significant reduction in MMP-8 levels following BP consumption (*p* = 0.028), which is important in the improvement of CV-related comorbidities (Table 3).

### 3.4. Functional Enrichment Analysis of Proteomics Data

**Figure 3:**
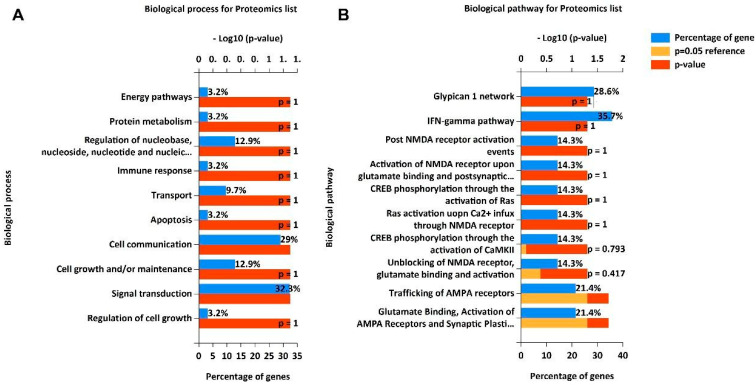
FunRich analysis. The possible (**A**) biological processes and (**B**) biological pathways performed on plasmatic proteins identified by the proteomic analysis. FunRich: Functional Enrichment tool.

Functional enrichment analysis for biological processes of proteins revealed that most of the proteins are involved in signal transduction (32.3%) and cell communications (29%) followed by regulation of nucleobase, nucleoside, nucleotide and nucleic acid metabolism (12.9%), cell growth and/or maintenance (12.9%), and lipid transport (9.7%). The top 10 enriched biological processes are depicted in Figure 2A. Pathway analysis of these proteins identified enriched biological pathways, including the IFN gamma pathway (35.7%), glypican 1 network (28.6%), trafficking of AMPA receptors (21.4%), and Ras activation through NMDA receptor (14.3%) pathways (Figure 3B). Analysis for cellular component localizations revealed that most of the proteins are localized to the nucleus (64.3%) and cytoplasm (57.1%), followed by the plasma membrane (39.3%) and exosomes (21.4%) (Supplementary Figure S4).

### 3.5. Analysis of Protein–Protein Interactions (PPIs) via STRING

**Figure 4:**
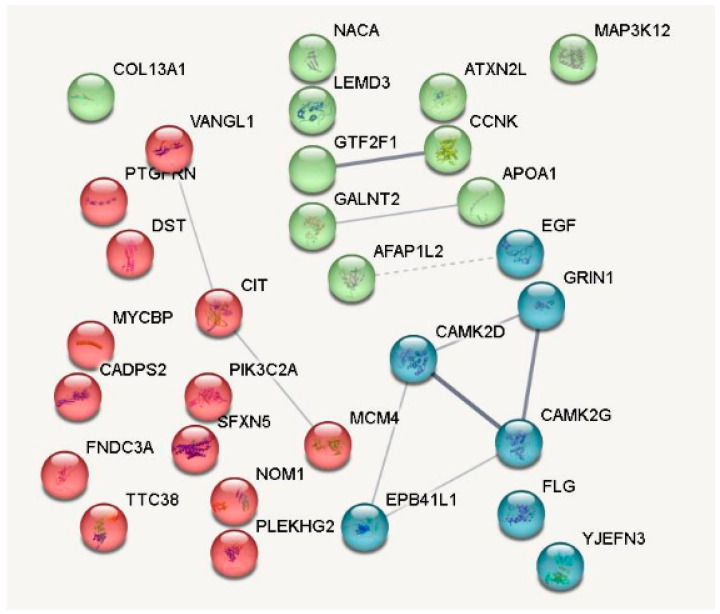
Protein–protein interactions and clusters of identified proteins by proteomic approaches (STRING database). The figure highlights the connections between differentially represented proteins. Proteins include collagen type XIII alpha 1 chain (COL13A1), Mitogen-activated protein kinase 12 (MAP3K12), Apolipoprotein A1 (APOA1), Epidermal Growth Factor (EGF), Phosphatidylinositol-4-Phosphate 3-Kinase (PIK3C2A), among others. Nodes represent proteins while the association between proteins are indicated by lines.

The STRING database was employed to unravel the intricate web of PPIs on the BP under study (Figure 4). The PPI network generated revealed several noteworthy clusters (signal transduction, cell growth, and lipid transport), indicative of potential functional conglomerates in CV health and disease.

Within the network, a prominent cluster was identified, encompassing proteins such as MAP3K12, a kinase implicated in signal transduction. This cluster also included CCNK, which is involved in cell cycle regulation, hinting at a possible collective role in cellular proliferation and maintenance. Another significant interaction was observed between PIK3C2A, a member of the phosphoinositide 3-kinase family, and NOM1, suggesting a link to cellular growth pathways. The network further identified a linkage between CIT, a protein kinase involved in the regulation of the actin cytoskeleton, and MYCBP, which is known to associate with the c-Myc proto-oncogene product, a factor essential for cellular growth and division. Additionally, proteins such as EGF, a key molecule in cell growth, proliferation, and differentiation, and APOA1, the major protein component of high-density lipoprotein (HDL) in plasma, were depicted. The network also depicted FLG, involved in skin barrier function, and GRIN1, which encodes a subunit of the N-methyl-D-aspartate (NMDA) receptor. Clusters involving proteins related to the extracellular matrix, such as COL13A1 and DST, were noted, implying a potential regulatory impact on tissue remodelling and integrity. Furthermore, proteins with a role in the ubiquitin–proteasome system, such as YJEFN3, were also part of the network, reflecting the comprehensive nature of the BP’s influence on cellular mechanisms. The PPI analysis through the STRING tool thus provides a panoramic view of the potential biological effects of BP, highlighting their diverse influence on various cellular pathways and processes. This intricate network of interactions lays the foundation for understanding the molecular basis of the peptides’ therapeutic potential.

## 4. Discussion

BPs have emerged as a molecule of profound interest due to their potential therapeutic properties [1]. The beneficial effect of BP from dry-cured pork ham over the CV system has been investigated in two clinical trials from our group [19,20], although few mechanistic studies have been designed to explore the wider range of activities of such BP in the CV system [23].

Indeed, the observed modulation of gene expression by BP, particularly in C-HUVECs, could have significant implications for CV health. Indeed, *MT-RNR1* and *MT-RNR2*, which encode mitochondrial ribosomal RNAs, have been implicated in the regulation of insulin sensitivity and metabolic homeostasis, both of which are critical factors in the aetiology of CV diseases [28]. The upregulation of these genes following treatment with BP in C- and GD-HUVECs may suggest a beneficial role in mitochondrial function and energy metabolism, which are crucial in maintaining CV health. Moreover, the upregulation of *MMP-1* might also indicate an active role in vascular remodelling processes, which are essential for the maintenance of vascular integrity [29,30,31]. Conversely, the downregulation of genes such as *TGF-βI*, associated with cell adhesion and migration, and *PTX3*, an acute-phase protein linked to inflammation and CV risk, suggests that BP may elicit its beneficial effects through the modulation of these signaling pathways [32,33]. When we analysed the signaling level, Western blotting revealed a significant reduction in NF-κB phosphorylation following the combined administration of TNF-α and BP, consistent with previous findings from our group in transfected cells [22]. However, transcriptomic analysis failed to demonstrate a corresponding reversal of inflammatory and oxidative gene expression compared to the TNF-α stimulated condition, suggesting the existence of additional mechanisms beyond NF-κB phosphorylation.

In pursuit of clarifying the advantageous effects of BP on endothelial integrity in vitro, we examined two pivotal proteins associated with improvements in endothelial cell function: *p*-ERK and *p*-AKT [34]. It is known that certain synthetic organic compounds, implicated in various diseases, can increase ERK phosphorylation [35], while inhibiting this pathway may contribute to plaque stability [36], paralleling our findings of reduced *p*-ERK levels following BP treatment. In the context of AKT, its phosphorylation state is known to be diminished by oxidized LDL, a contributor to CV diseases [37], whereas increased phosphorylation of AKT in the nucleus is associated with cardioprotective effects [38], reinforcing our observations of enhanced AKT phosphorylation in BP treatment.

STRING analysis of DEGs in C- and GD-HUVECs identified several proteins that have direct implications in CV disease, forming a complex network of interactions that underlie key pathophysiological processes of endothelial dysfunction. Genes such as *E-selectin*, *MMP-1*, *FGF5*, and *SERPIND1* play a pivotal role in the inflammatory and oxidative pathways, facilitating the adhesion of leukocytes, degradation of interstitial collagens or angiogenesis, and the coagulation cascade [30,31,39,40]. The dysregulation of these genes in GD-HUVECs underlies the vascular complications commonly observed in diabetes, providing a rationale for targeted therapeutic interventions such as BP to ameliorate CV outcomes in pathological conditions. However, the GD-HUVECs’ response to BP was not sufficient to account for the observed phenotypic changes, indicating their inability to fully revert the pathological phenotype. Nevertheless, other peptides such as PEP1 from rice and KQS-1 from adzuki bean have been suggested as potential natural anti-inflammatory and antioxidant therapies after transcriptomic analysis of cultured cells [41,42]. These findings emphasize the complexity of cellular responses and underscore the varying sensitivity of C-HUVECs and GD-HUVECs to these treatments (BP and HT) and the need for a comprehensive understanding of the multiple signaling pathways involved. Collectively, these results suggest that BP may be more effective in preventive applications rather than in therapeutic interventions. As a positive control, HT was able to reverse the pro-inflammatory and pro-oxidant effect of TNF-α, thus confirming previous results about the protective effects of HT against oxidative stress in HUVECs [43]. HT significantly reduced reactive oxygen species (ROS) levels in these cells, thereby enhancing cell viability and function. This antioxidant action is vital, as oxidative stress is a key factor in endothelial dysfunction, which can lead to cardiovascular diseases. According to a previous study, and confirming our results, HT treatment reduced the expression of endothelial adhesion molecules, such as *ICAM-1* and *VCAM-1*, which are important mediators of vascular inflammation [44]. This suggests that HT could play a role in preventing atherosclerosis by inhibiting leukocyte adhesion to the endothelium. Furthermore, it is of paramount importance to highlight that the effects of HT on HUVECs were previously analysed in several studies; however, the effects of both BP and HT on GD-HUVECs have not been studied elsewhere. This way, our results open the possibility of further analysing the differences between C- and GD-HUVECs.

Following the in vitro studies, our focus aimed to decipher the underlying molecular mechanisms and biological pathways influenced by BP intake in patients with CV risk [19]. Notably, the FunRich analysis of our plasma proteomic data [27] highlighted the involvement of inflammatory and oxidative pathways, including the Ras protein, which is intricately linked to the secretion of pro-inflammatory cytokines IL-6 and IL-8. Conversely, Ras inhibition correlated with a downregulation of these interleukin genes [45]. Additionally, the MAPK pathway emerged as a promising therapeutic target [46], aligning with our in vitro findings that implicate ERK —a component of the MAPK cascade—in the modulation of our experimental outcomes [47].

These in vitro findings indicate that BP treatment modulated the expression of genes and proteins involved in vascular stability and plaque formation, such as *MMP-1*, *TGF-β1*, and *p*-ERK, which are known to influence vascular integrity [29,32,36]. Additionally, the regulation of *MT-RNR1* and *MT-RNR2* suggests a potential role in modulating insulin sensitivity and glucose homeostasis [28]. The preservation of vascular function might also be mediated by the reduction in inflammatory markers such as *PTX3*, which further contributes to the reduction in the CV risk [33]. However, these protective effects were limited in our GD-HUVECS model, representing a pathological condition. Given that these compounds (HT and BP) alone are insufficient to reverse established pathology, we extended our investigation to evaluate the potential of BP as a preventive intervention in volunteers at CV risk but not yet exhibiting pathological features.

Moreover, the quantitative plasma protein expression provided by the proteomic analysis (ScaffoldQ software) of the human clinical study revealed differential expression of CV-relevant proteins, including a significant increase in APOA1 and a decrease in APOA2, post-consumption of BP-enriched ham. These findings suggest a beneficial modulation of lipid transport and metabolism, which are critical factors in CV health. Our findings align with the nuanced perspective presented by Remaley et al. [48], suggesting that decreased levels of APOA2 may confer a cardioprotective effect. Contrastingly, the increase in APOA1 levels observed in our study corroborates its established role in promoting CV health through mechanisms such as LDL oxidation inhibition, facilitating the clearance of toxic metabolites, and exerting anti-inflammatory and antioxidant effects [49,50]. The APOB/APOA1 ratio post-treatment, which reflects the balance of proatherogenic to antiatherogenic lipoproteins, was reduced in our study post-treatment. This decrease is supported by the literature as indicative of a reduced risk for atherosclerotic events, thereby endorsing the CV benefits of the BP intervention [51,52]. Matrix metalloproteinases, particularly MMP-8, have been implicated in CV pathophysiology, predominantly associated with unstable atherosclerotic plaque phenotypes [53,54]. By our transcriptomic data (*MMP-1*), a significant reduction in MMP-8 levels following BP consumption suggests a stabilizing effect on vascular integrity, potentially mitigating the progression of atherosclerosis. These data nicely complement the cardiometabolic improvement found by the regular consumption of BP-enriched ham [19]. Interestingly, proteomics in in vitro experiments revealed that the BP influenced various biochemical pathways in the cells [55], but further in vivo proteomics investigations, such as the current study, are needed to estimate the clinical relevance of this intake.

The in vitro models used in this study, which have been previously established and optimized by our group [2,25], provide a reliable method to study the potential of compounds from vegetal and animal origin in distinct scenarios resembling those found in chronic endothelial dysfunction. In addition, we included in this study a treatment with HT as a positive control, as it is considered a gold standard of antioxidant bioactive compound from plant origin [56,57] and can reverse the TNF-α effect, particularly in C-HUVECs. The combination of transcriptomic and proteomic techniques, along with advanced computational tools, has become a cornerstone for understanding how bioactive compounds interact with biological systems [58,59,60]. These methods are further strengthened by molecular approaches like qPCR, Western blotting, and ELISA, which help validate the specific targets identified in broader omics analyses [61,62]. In this study, we used these tools to investigate the pathways influenced by BP, both in vitro and in vivo, finding alignment between cellular models of early endothelial dysfunction and clinical data. While the findings shed light on how BP can impact endothelial health and cardiovascular function, they also highlight the complexity of these molecular interactions. Moving forward, it will be important to expand the use of these techniques to explore the effects of BPs in a wider range of pathological conditions and to confirm their therapeutic potential in larger, more diverse populations. Future assays measuring mitochondrial function are granted to elucidate the improvements in mitochondrial bioenergetics affected by both treatments. The use of only three donors per group in the in vitro assay, while a limitation, actually adds robustness to our study by demonstrating consistent treatment effects despite inherent biological variability among primary cells. By adopting this integrated approach, we can better understand and predict how these compounds might support CV health.

## 5. Conclusions

A thorough molecular analysis of the impact of BP from dry-cured ham on CV health, assessing endothelial cells from both healthy and GD pregnancies, was performed. The identification of pathways involved in atherosclerosis risk, as revealed by transcriptomic and proteomic analysis, further supports the potential of BPs to exert a preventive effect against vascular pathology.

To the best of our knowledge, this is the first study reporting the cardioprotective effects of BP by affecting lipoproteins and MMPs. Our data are in agreement with those reported by others who have shown the potential of HT as an antioxidant agent, and serve to confirm the utility of such cellular models. These findings also reveal distinctions between mechanisms underlying the effects of BP and HT. By increasing an understanding of how BPs affect signaling proteins such as *p*-ERK, *p*-AKT, and *p*-NF-κB, this study highlights the potential of these peptides as modulators of CV health. Moving forward, further exploration of their molecular mechanisms will be crucial to translating these discoveries into practical clinical applications, paving the way for new strategies in the prevention of cardiovascular diseases.

## Figures and Tables

**Table 1:** Profiling of the specific and significant changes after bioactive peptide treatment (n = 3), in HUVECs, determined by differentially expressed genes (DEGs).

C-HUVECs: Control vs. BP Treatment				
**Gene Name**	Gene ID	Function	2log (BaseMeanB/ BaseMeanA)	Adjusted *p*-Value
*MT-RNR2*	ENSG00000210082	Protects endothelial cells from inflammation by suppressing oxidative stress	2.71	4.70 × 10^−75^
*MT-RNR1*	ENSG00000211459	Regulates insulin sensitivity and metabolic homeostasis	2.595	1.93 × 10^−25^
*KRT17*	ENSG00000128422	Expressed in nail bed, hair follicle, sebaceous glands, and other epidermal appendages	−3.767	4.01 × 10^−12^
*MX1*	ENSG00000157601	Antiviral activity against a wide range of RNA viruses and some DNA viruses	−2.354	3.39 × 10^−8^
*MMP-1*	ENSG00000196611	Breakdown of the extracellular matrix in normal physiological processes	1.301	2.23 × 10^−7^
*SULF1*	ENSG00000137573	The expression of this gene may be downregulated in several types of cancer.	−0.766	7.83 × 10^−7^
*TGFBI*	ENSG00000120708	Plays a role in cell adhesion	−1.453	1.08 × 10^−6^
*MIR205HG*	ENSG00000230937	RNA gene, affiliated with the IncRNA class	−3.914	1.08 × 10^−6^
*DSP*	ENSG00000096696	Form a component of functional desmosomes	−2.612	2.84 × 10^−5^
*KRT7*	ENSG00000135480	Blocks interferon-dependent interphase and stimulates DNA synthesis in cells	−0.765	0.0001
*PTX3*	ENSG00000163661	Plays a role in inflammatory reactions	−0.739	0.0005
*KRT14*	ENSG00000186847	Type I keratin	−2.765	0.0007
*COL3A1*	ENSG00000168542	Encodes pro-alpha1 chains of type III collagen	−0.721	0.0007
*SELE*	ENSG00000007908	Responsible for the accumulation of blood leukocytes at sites of inflammation	−1.261	0.0032
*CCDC190*	ENSG00000185860	Related to cannabis and hallucinogen dependence	−4.039	0.0038
*PKP1*	ENSG00000081277	Contributes to epidermal morphogenesis	−5.209	0.0113
*IFI44L*	ENSG00000137959	Facilitates inflammatory cytokine secretion	−1.927	0.0117
*PLXNA4*	ENSG00000221866	Enables semaphorin receptor activity	−0.556	0.0180
*TGFB2*	ENSG00000092969	Multifunctional protein that regulates various processes such as angiogenesis and heart development	−0.702	0.0197
*SULT1B1*	ENSG00000173597	May play a role in gut microbiota–-host metabolic interaction	−0.608	0.0222
*ANXA8*	ENSG00000286129	Acts as an anticoagulant that inhibits the thromboplastin-specific complex	-Inf	0.0264
*SOX18*	ENSG00000203883	Plays a role in hair, blood vessel, and lymphatic vessel development	0.622	0.0482
GD-HUVECs: Control vs. BP Treatment				
Gene Name	Gene ID	Function	2log (BaseMeanB/ BaseMeanA)	Adjusted *p*-Value
*MT-RNR1*	ENSG00000211459	Regulates insulin sensitivity and metabolic homeostasis	3.170	0.013
*H3C10*	ENSG00000278828	Proteins responsible for the nucleosome structure of the chromosomal fibre in eukaryotes	−3.196	0.021

Adjusted *p*-value is based on a fold change of >2 or <−2. Confluent C-HUVECs were stimulated for 6 h in the absence/presence of 300 µM BP. Total RNA was then extracted from cells and processed for RNA-SEQ. A positive result in 2log (BaseMeanB/BaseMeanA) represents an increased level following biopeptide treatment, while a negative result represents an increased level in the control treatment.

**Table 2:** Differentially expressed proteins in plasmatic samples identified by LC-MS, using Scaffold and Sequest software.

Protein Name (Using Scaffold Software)	Gene Name	*p*-Value
Capping protein regulator and myosin 1 linker 2	*CARMIL2*	<0.0001
Apolipoprotein AI	*APOA1*	<0.0001
Apolipoprotein AII	*APOA2*	<0.0001
Calcium-dependent secretion activator 2	*CADPS2*	<0.0001
Dystonin	*DST*	0.081
Zinc finger SWIM domain-containing protein 8	*ZSWIM8*	0.1
Collagen type XIII alpha 1 chain	*COL13A1*	0.0088
Nascent polypeptide-associated complex subunit alpha	*NACA*	0.16
Proline-rich 25	*PRR25*	0.39
Calcium/calmodulin-dependent protein kinase II gamma	*CAMK2G*	0.15
Glutamate ionotropic receptor NMDA type subunit 1	*GRIN1*	0.15
Cyclin K	*CCNK*	0.25
Nucleolar protein with MIF4G domain 1	*NOM1*	0.023
Calcium/calmodulin-dependent protein kinase II delta	*CAMK2D*	0.39
Filaggrin	*FLG*	0.023
Tetratricopeptide repeat domain 38	*TTC38*	0.39
ARHGAP23 (Fragment)	*ARHGAP23*	0.096
Erythrocyte membrane protein band 4.1 like 1	*EPB41L1*	0.39
Minichromosome maintenance complex component 4	*MCM4*	0.39
Sideroflexin 5	*SFXN5*	0.15
Protein Name (Using Sequest Software)	Gene Name	
MYC binding proteins	*MYCBP*	
Polypeptide N-acetylgalactosaminyltransferase 2	*GALNT2*	
General transcription factor IIF subunit 1	*GTF2F1*	
VANGL planar cell polarity protein 1	*VANGL1*	
Fibronectin type III domain containing 3A	*FNDC3A*	
YjeF N-terminal domain containing 3	*YJEFN3*	
Prostaglandin F2 receptor inhibitor	*PTGFRN*	
Mitogen-activated protein kinase 12	*MAP3K12*	
Phosphatidylinositol-4-phosphate 3-kinase catalytic subunit type 2 alpha	*PIK3C2A*	
Epidermal growth factor	*EGF*	
Pleckstrin homology and RhoGEF domain containing G2	*PLEKHG2*	
Citron rho-interacting serine/threonine kinase	*CIT*	
LEM domain containing 3	*LEMD3*	
Parking RBR E3 ubiquitin protein ligase	*PRKN*	
Myosin heavy chain 16 pseudogene	*MYH16*	
Ataxin 2 like	*ATXN2L*	
Actin filament-associated protein 1-like 2	*AFAP1L2*	

**Table 3:** Changes in identified protein levels measured with ELISA.

(n = 30)	Before BP Consumption	After BP Consumption	*p*-Value
APOA1	0.8248	0.8560	0.361
(ng/mL)	[0.567–1.0884]	[0.6944–1.0899]	
APOA2	0.1623	0.1591	0.862
(ng/mL)	[0.1434–0.1750]	[0.1513–0.1741]	
APOB	0.4945	0.4534	0.936
(ng/mL)	[0.3511–0.5983]	[0.3192–0.6799]	
MMP-8	0.4845	0.4521	0.028 *
(pg/mL)	[0.4196–0.5681]	[0.3806–0.5174]	
APOB/APOA1	0.5303	0.4792	0.018 *
	[0.3879–0.7070]	[0.3582–0.6559]	

Data are presented as median [interquartile range] for non-normally distributed variables. Comparisons are assessed by the Wilcoxon test. Significant *p*-values were expressed by bold formatting and the * symbol.

## Data Availability

Data is contained within the article and Supplementary Material.

## Supplementary Materials

The following supporting information can be downloaded at: https://www.mdpi.com/article/10.3390/antiox14070772/s1, Table S1. Profiling of the specific and significant changes to hydroxytyrosol (n = 3), in HUVECs determined by differentially expressed genes (DEGs). Figure S1. Relative expression of TNF-α and VCAM-1 in A) C-HUVECs and B) GD-HUVECs. TNF-α expression was measured after 1 ng/mL TNF-α treatment for 6 h and/or 100 µM HT. Fold induction for each gene was calculated versus control at the corresponding time point. Results represent the average ± SD of three independent experiments. Statistical significance was determined via ANOVA followed by Tukey’s multiple comparison test. (*) Significantly different from the control. *: *p*-value < 0.05; **: *p*-value < 0.01; ***: *p*-value < 0.005. Figure S2. Protein expression levels of p-AKT and p-ERK were analysed by Western blot in C-HUVECs and GD-HUVECs. Protein expression was measured after 1 ng/mL TNF-α treatment for 6 h and/or 100 µM HT. Figure S3. Differentially expressed genes (DEGs) in 1 ng/mL TNFα compared to TNFα + 300 µM BP. (A) DEG volcano plot of C-HUVECs. (B) DEG volcano plot of GD-HUVECs. Figure S4. FunRich analysis shows the possible cellular localization of the proteins. FunRich: Functional Enrichment tool.

## Abbreviations

ACE	Angiotensin-converting Enzyme.
AKT	Protein Kinase B.
APO	Apolipoprotein.
BP	Bioactive Peptides.
COL13A1	Collagen Type XII Alpha Chain.
CV	Cardiovascular.
DGE	Differential Gene Expression.
DTT	Dithiothreitol.
EGF	Epidermal Growth Factor.
ELISA	Enzyme-Linked Immunosorbent Assay.
ERK	Extracellular Signal-Regulated Kinase.
FBS	Fetal Bovine Serum.
FPKM	Fragments Per Kilobase of Exon Per Million Mapped Fragments.
GADPH	Glyceraldehyde 3-phosphate Dehydrogenase.
GD	Gestational Diabetes.
GO	Gene Ontology.
HDL	High-density Lipoprotein.
HMG-CoAR	3-hydroxy-3-methyl-glutaril-CoA Reductase.
HT	Hydroxytyrosol.
HUVECs	Human Umbilical Vein Endothelial Cells.
KEGG	Kyoto Encyclopedia of Genes and Genomes.
LC-MS	Liquid Chromatography/Mass Spectometry.
MAP3K12	Mitogen-activated Protein Kinase 12.
MMP	Matrix Metalloproteinase.
MT-RNR1	Mitochondrially Encoded 12S RNA.
NMDA	N-methyl-D-Aspartate.
PIK3C2A	Phosphatidylinositol-4-Phosphate-3-kinase.
PPIs	Protein–protein interactions.
q-PCR	Quantitative PCR.
ROS	Reactive Oxygen Species.
SDS-PAGE	Sodium Dodecyl Sulfate-polyacrylamide Gel Electrophoresis.
TNF-α	Tumour Necrosis Factor-α.
VCAM-1	Vascular Cell Adhesion Molecule-1.
