# Supplementary material for: Cardioprotective Peptides from Dry-Cured Ham in Primary Endothelial Cells and Human Plasma: An Omics Approach
Source: Antioxidants (Basel). 2025 Jun 24;14(7):772. doi: 10.3390/antiox14070772 (PMC12291938; doi:10.3390/antiox14070772)
Supplement: Supplementary file 1 [file antioxidants-14-00772-s001.zip › antioxidants-3680113-supplementary.pdf]

SUPPLEMENTAL DATA

METHODOLOGY

Cell Viability Assay for Hydroxytyrosol Treatment

Cells were required to reach a confluency of 60-70%, and 100 µL of cells per well were seeded in 96-well plates at a concentration of 30,000 cells per well. The cells were then incubated at 37 °C for 24 hours. After this period, they were treated with varying concentrations of HT: 1, 5, 10, 20, 50, 100, 500, and 1000 µM. The treatment was incubated at 37 °C for another 24 hours. Subsequently, 30 µL of thiazolyl blue tetrazolium bromide (MTT) at a concentration of 1.9 mg/mL was added, followed by a 4-hour incubation at 37 °C in the dark. The medium was then aspirated, and 200 µL of dimethyl sulfoxide (DMSO) per well was added, with a 30-minute incubation at room temperature under orbital agitation. Finally, the plate was measured at 570 nm with a reference wavelength of 620 nm. A non-toxic concentration of 100 µM was used for *in vitro* studies.

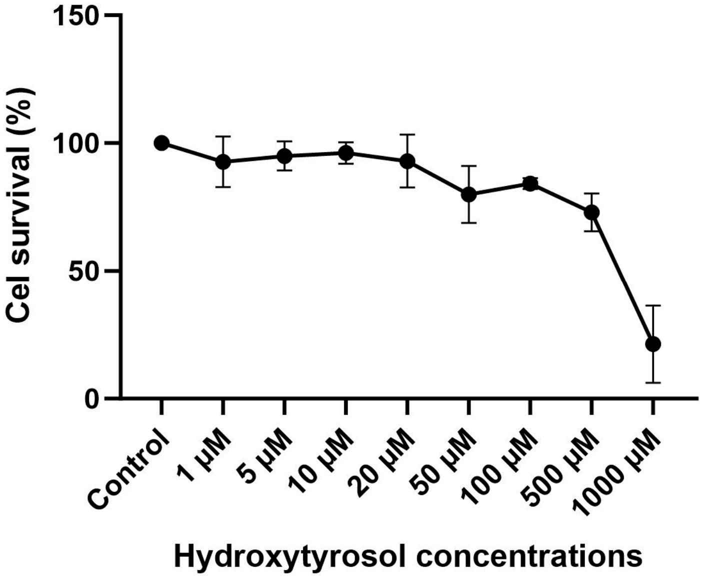

SUPPLEMENTARY TABLES

Supplementary Table S1. Profiling of the specific and significant changes to hydroxytyrosol (n=3), in HUVECs determined by differentially expressed genes (DEGs).

		**C-HUVECs: Control vs BP treatment**		
**Gene Name**	**Gene ID**	**Function**	**2log (baseMeanB / BaseMeanA)**	**Adjusted P-value**
STC1	ENSG00000159167	Stimulates renal phosphate reabsorption, and could therefore prevent hypercalcemia	3.3298	5,33E+06
MT-RNR1	ENSG00000211459	Regulates insulin sensitivity and metabolic homeostasis	3.713	6,56E+06
HMOX1	ENSG00000100292	Exhibits cytoprotective effects	2.677	0.0002
MT-TQ	ENSG00000210107	Transfers glutamine to a growing polypeptide chain at the ribosome site of protein synthesis during translation	4.455	0.0002
MT-RNR2	ENSG00000210082	Protects endothelial cells from inflammation by suppressing oxidative stress	0.268	0.0003
GAS6-AS1	ENSG00000233695	RNA gene affiliated with the IncRNA class	0.307	0.0009
MT-TS1	ENSG00000210151	Transfers serine to a growing polypeptide chain at the ribosome site of protein synthesis during translation	4.617	0.0026
MT-ND6	ENSG00000198695	NADH-ubiquinone oxidoreductase chain 6	2.728	0.0034
H1-3	ENSG00000124575	Histone 1 part from the macromolecular structure known as the chromatin fibre	-2.942	0.0048
SOX18	ENSG00000203883	Plays an essential role in embryonic	-3.185	0.0104

		cardiovascular development		
H2BC18	ENSG00000203814	Histone H2B, core component of the nucleosome	-2.636	0.0152
H3C12	ENSG00000197153	Histone H3.1, core component of the nucleosome	-2.558	0.0190
H3C3	ENSG00000287080	H3 clustered histone 3, core component of the nucleosome	-2.528	0.0314
RN7SL1	ENSG00000276168	Cytoplasmic ribonucleoprotein complex	-2.037	0.0321

GD-HUVECs: Control vs HT treatment

Gene Name	Gene ID	Function	2log (baseMean B / BaseMean A)	Adjusted P-value
STC1	ENSG00000159167	Stimulates renal phosphate reabsorption, and could therefore prevent hypercalcemia	3.889	2,20E+04
HMOX1	ENSG00000100292	Exhibits cytoprotective effects	3.083	6,37E+05
ADAMTS 4	ENSG00000158859	May be involved in the turnover of aggrecan, a cartilage proteoglycan	2.775	0.002
EXOC3L2	ENSG00000283632	Belongs to the SEC6 family	2.794	0.002
SERPINB2	ENSG00000197632	Belongs to the serping family	0.199	0.005
NR4A1	ENSG00000123358	May inhibit NF-kB transactivation of IL2	3.495	0.042
SOCS1	ENSG00000185338	Negative regulates cytokines that signal through the JAK/STAT3 pathway	3.873	0.042

Adjusted P-value is based on a fold change of > 2 or <−2.
Confluent C-HUVECs were stimulated for 6 h in the absence/presence of 300 µM BP. Total RNA was then extracted from cells and processed for RNA-SEQ.
A positive result in 2log (BaseMeanB/BaseMeanA) represents an increased level following HT treatment while a negative result represents an increased level in control treatment.

SUPPLEMENTARY FIGURES

Supplementary Figure S1. Relative expression of TNF-α and VCAM-1 in A) C-HUVECs and B) GD-HUVECs. TNF-α expression was measured after 1 ng/mL TNF-α treatment for 6 hours and/or 100 μM HT. Fold induction for each gene was calculated versus control at the corresponding time point. Results represent the average ± SD of three independent experiments. Statistical significance was determined via ANOVA followed by Tukey's multiple comparison test. (*) Significantly different from the control. *: p-value<0.05; **: p-value<0.01; ***: p-value<0.005.

Supplementary Figure S2. Protein expression levels of p-AKT and p-ERK were analysed by Western blot in C-HUVECs and GD-HUVECs. Protein expression was measured after 1 ng/mL TNF-α treatment for 6 hours and/or 100 μM HT.

Supplementary Figure S3. Differentially expressed genes (DEGs) in 1 ng/mL TNFα compared to TNFα + 300 μM BP. A) DEG volcano plot of C-HUVECs. B) DEG volcano plot of GD-HUVECs.

Supplementary Figure S4. **FunRich analysis shows the possible cellular localization of the proteins**. FunRich: Functional Enrichment tool.

FIGURES

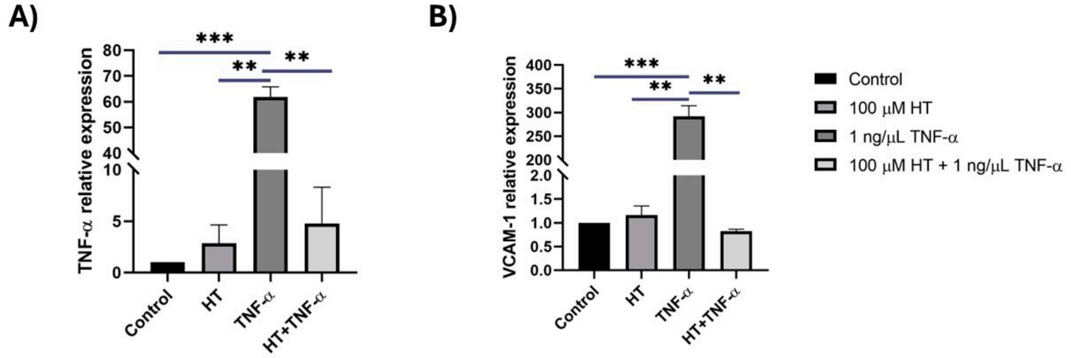

Supplementary Figure S1. Noguera-Navarro *et al.* 2025.

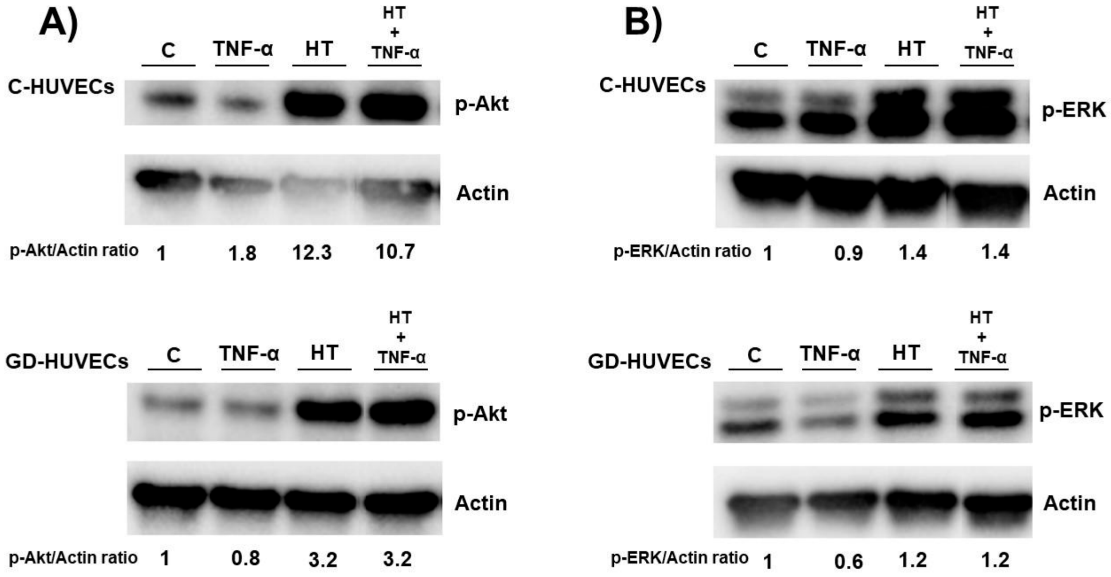

Supplementary Figure S2. Noguera-Navarro *et al.* 2025.

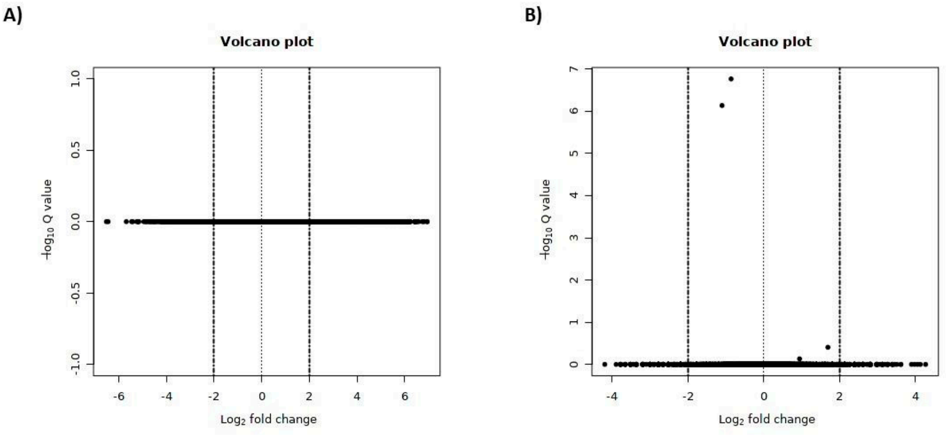

Supplementary Figure S3. Noguera-Navarro *et al.* 2025.

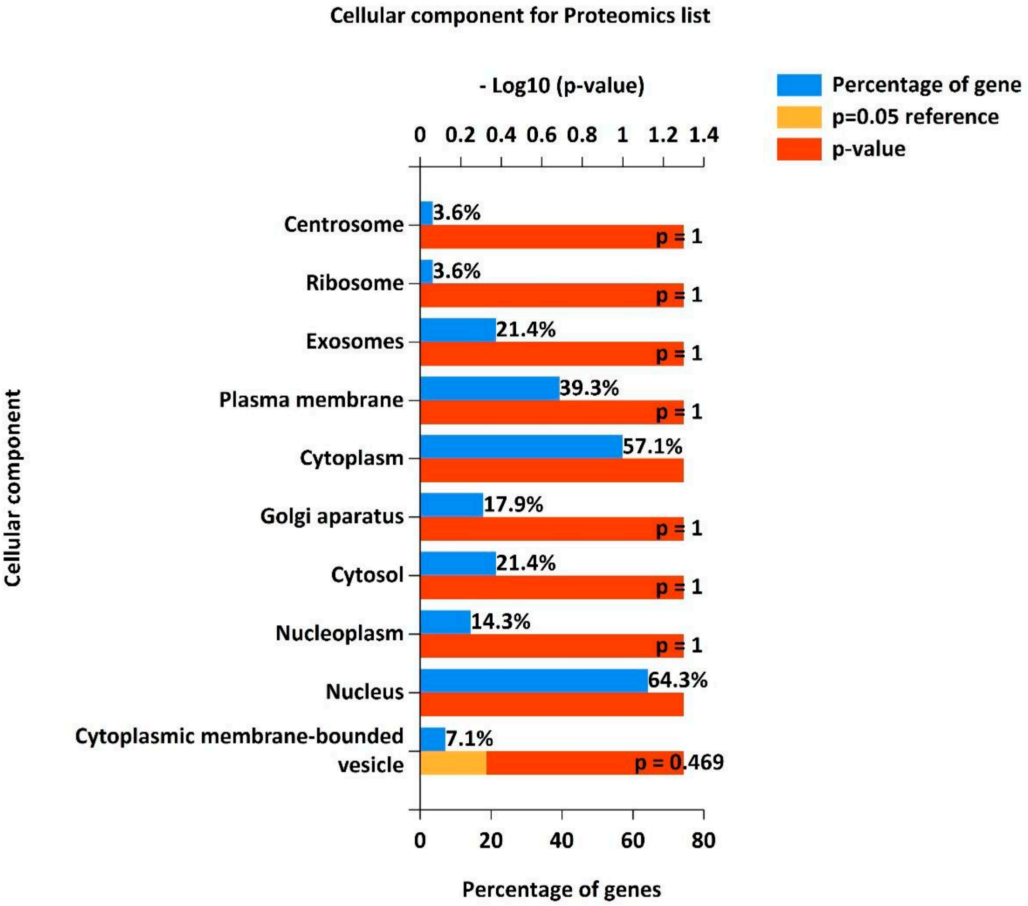

Supplementary Figure S4. Noguera-Navarro *et al.* 2025.
